# Optimized electrocardiographic criteria for the detection of left ventricular hypertrophy in obesity patients

**DOI:** 10.1002/clc.23333

**Published:** 2020-01-28

**Authors:** Sanne M. Snelder, Sweder W.E. van de Poll, Lotte E. de Groot – de Laat, Isabella Kardys, Felix Zijlstra, Bas M. van Dalen

**Affiliations:** ^1^ Department of Cardiology Franciscus Gasthuis and Vlietland Rotterdam The Netherlands; ^2^ Department of Cardiology Maasstad Ziekenhuis Rotterdam The Netherlands; ^3^ Department of Cardiology, Thoraxcenter University Medical Center Rotterdam, Erasmus MC Rotterdam The Netherlands

**Keywords:** cornell voltage, electrocardiogram, left ventricular hypertrophy, obesity/obese, Peguero‐Lo Presti criteria, Sokolow‐Lyon index

## Abstract

**Background:**

Despite a generally high specificity, electrocardiographic (ECG) criteria for the detection of left ventricular hypertrophy (LVH) lack sensitivity, particularly in obesity patients.

**Objectives:**

The aim of the study was to evaluate the accuracy of the most commonly used ECG criteria (Cornell voltage and Sokolow‐Lyon index), the recently introduced Peguero‐Lo Presti criteria and the correction of these criteria by body mass index (BMI) to detect LVH in obesity patients and to propose adjusted ECG criteria with optimal accuracy.

**Methods:**

The accuracy of the ECG criteria for the detection of LVH was retrospectively tested in a cohort of obesity patients referred for a transthoracic echocardiogram based on clinical grounds (test cohort, n = 167). Adjusted ECG criteria with optimal sensitivity for the detection of LVH were developed. Subsequently, the value of these criteria was prospectively tested in an obese population without known cardiovascular disease (validation cohort, n = 100).

**Results:**

Established ECG criteria had a poor sensitivity in obesity patients in both the test cohort and the validation cohort. The adjusted criteria showed improved sensitivity, with optimal values for males using the Cornell voltage corrected for BMI, (RaVL+SV3)*BMI ≥700 mm*kg/m^2^; sensitivity 47% test cohort, 40% validation cohort; for females, the Sokolow‐Lyon index corrected for BMI, (SV1 + RV5/RV6)*BMI ≥885 mm*kg/m^2^; sensitivity 26% test cohort, 23% validation cohort.

**Conclusions:**

Established ECG criteria for the detection of LVH lack sufficient sensitivity in obesity patients. We propose new criteria for the detection of LVH in obesity patients with improved sensitivity, approaching known sensitivity of the most commonly used ECG criteria in lean subjects.

## 1 INTRODUCTION

The prevalence of obesity has increased rapidly, and nowadays more people are obese than underweight.^1^ Left ventricular hypertrophy (LVH) occurs frequently in obesity patients, even in the absence of comorbidities such as hypertension^2^, ^3^ and is associated with increased risk of cardiovascular disease, morbidity, and mortality.^4^, ^5^, ^6^ Although echocardiography is a more sensitive tool to identify LVH, the standard electrocardiogram (ECG) remains widely used, because of its established clinical value, broad availability, and low costs.^7^ ECG criteria for the diagnosis of LVH have been used since 1914.^8^ Nowadays, the two most commonly used ECG criteria are the Cornell voltage^9^ and the Sokolow‐Lyon index.^10^ Despite a generally high specificity, most ECG criteria for LVH lack sensitivity.^11^ The value of these criteria is particularly questionable in obesity patients^12^, ^13^ because obesity is responsible for geometrical and electrophysiological changes of the heart and ECG voltages may be attenuated by subcutaneous adipose tissue.^14^, ^15^ Recently, Peguero and Lo‐Presti introduced more sensitive ECG criteria for the detection of LVH.^16^ Until now, these criteria have not been specifically tested in obese subjects. Finally, Angeli et al.^17^ introduced a correction to the Cornell voltage by body mass index (BMI) to improve the performance of traditional ECG criteria.

The aim of the current study was to retrospectively evaluate the accuracy of various ECG criteria to detect LVH in obesity patients and to propose adjusted ECG criteria with optimal sensitivity for this group of patients. Subsequently, the identified optimal criteria were prospectively tested in an obese population without suspicion of or known cardiovascular disease.

## 2 METHODS

The accuracy of the Cornell voltage, Sokolow‐Lyon index, Peguero‐Lo‐Presti criteria, and the correction of Cornell voltage by BMI for detection of LVH was retrospectively tested in obesity patients who were referred for a transthoracic echocardiogram based on clinical grounds (suspicion on or history of cardiovascular disease) (test cohort). From these data, ECG criteria with optimal sensitivity for the detection of LVH were developed by adjusting the cut‐off values and correcting all voltage criteria for BMI. After this, the value of these criteria was prospectively tested in an obese population without suspicion of or history of cardiovascular disease (validation cohort).

### 2.1 Test cohort

All obesity patients (BMI ≥35 kg/m^2^) who came to the Franciscus Gasthuis and Vlietland (Rotterdam, the Netherlands) in 2017 and underwent both an ECG and transthoracic echocardiography were included in the analysis. Patients with conditions potentially affecting the ECG voltage amplitude, such as a left or right bundle branch block, a paced rhythm, and imaging evidence of myocardial infarction or pericardial effusion, were excluded.

### 2.2 Validation cohort

All obese participants of the CARDIOBESE study^18^ were included for the validation cohort. In short, the CARDIOBESE study was designed to detect early signs of cardiac dysfunction in obesity patients without a suspicion of or known cardiovascular disease. Patients with a BMI of ≥35 kg/m^2^ scheduled for bariatric surgery were included. All research data acquisition was approved by the local research ethics committee and informed written consent was obtained from each participant.

### 2.3 ECG recording and analysis

**Figure 1:**
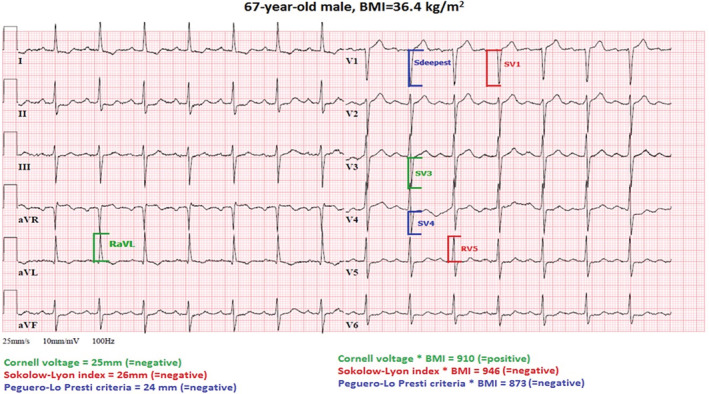
Electrocardiogram of a 67‐year‐old male obesity patient that meets the criteria for left ventricular hypertrophy based on the adjusted Cornell voltage*BMI, (RaVL+SV3)*BMI ≥700 mm*kg/m^2^. The diagnosis of left ventricular hypertrophy was confirmed by an echocardiogram. Note that none of the other criteria were positive. BMI, body mass index

A standard 12‐lead ECG was recorded at a paper speed of 25 mm/s and an amplification of 10 mm/mV. Heart rate, QRS duration, R‐wave and S‐wave heights, and QRS axis were measured. Left axis deviation was determined as QRS axis between −30° and −90°. Measurements were taken to the nearest 1 mm. The most commonly used ECG criteria were analyzed (Figure 1): the Cornell voltage, RaVL + SV3 (considered positive ≥28 mm in male subjects and ≥ 20 mm in female subjects)^9^; Sokolow‐Lyon index, SV1 + RV5/RV6 (RV5 or RV6, whichever is greater; considered positive ≥35 mm)^10^; the Peguero‐Lo Presti criteria, SV4 + Sdeepest (considered positive ≥28 mm in males and ≥ 23 mm in females)^16^; and the correction of Cornell voltage by BMI, (RaVL + SV3)*BMI (considered positive ≥604 mm*kg/m^2^).^17^ Finally, we multiplied the Sokolow‐Lyon index and Peguero‐Lo Presti criteria by BMI.

### 2.4 Echocardiography

Two‐dimensional (2D) grayscale harmonic images were obtained in the left lateral decubitus position using a commercially available ultrasound system (EPIQ 7, Philips, Best, the Netherlands), equipped with a broadband (1‐5 MHz) X5‐1 transducer. All acquisitions and measurements were performed according to current guidelines.^19^, ^20^ Estimation of left ventricular mass (LVM) as determined by echocardiography was used as the golden standard. Interventricular septal thickness (IVSd), posterior wall thickness (PWd), and left ventricular dimension (LVEDD) were all measured at end diastole. The LVM was calculated according to the Deveraux formula using these measurements: LVM (g) = 0.80 × {1.04[(IVSd + LVEDD + PWd)^3^‐(LVEDD)^3^]} + 0.6. LVM was abnormal if LVM ≥225 g for males and ≥163 g for females. The LVM was divided by the body surface area (BSA) to calculate the LVM‐index (LVMI). BSA was calculated by the Mosteller formula.^21^ LVH was defined as LVMI ≥102 g/m^2^ for males and ≥88 g/m^2^ for females.^19^

### 2.5 Statistical analysis

To compare baseline characteristics between the two cohorts, the Student *t* test was used for continuous variables and the *χ* ^2^ test for categorical variables. Continuous values were expressed as mean ± SD and categorical values as percentages. The sensitivity, specificity, positive predictive values (PPV) and negative predictive values (NPV), and their 95% confidence intervals were calculated for the ECG criteria in both groups. Differences in sensitivity and specificity between cohorts were tested with the *χ* ^2^ test. A two‐tailed *P* value <.05 was considered statistically significant. Optimal cut‐off values for all ECG criteria were manually calculated for both genders by a receiver operating characteristic (ROC) curve, using a fixed specificity of 95%.^10^, ^14^, ^22^, ^23^ Area under the curve (AUC) was calculated. Statistical analyses were performed with SPSS version 25.0 or higher (SPSS Inc., Chicago).

## 3 RESULTS

### 3.1 Patient characteristics

A total of 194 patients were included in the test cohort and 100 in the validation cohort. Twenty‐seven patients were excluded in the test cohort; five patients because of a paced rhythm, 15 patients because of a bundle branch block, and seven patients showed wall motion abnormalities on echocardiography. No patients were excluded due to poor echocardiographic windows.

Patients in the test cohort were older, had a higher heart rate and more comorbidities such as diabetes mellitus and hypertension (Table 1). Patients in the validation cohort had a higher BSA (2.3 ± 0.2 vs 2.5 ± 0.2 m^2^, *P* < .001) compared to the test cohort. LVM (197 ± 67 vs 196 ± 63 g, *P* = .83), and LVMI (94 ± 81 vs 79 ± 22 g/m^2^, *P* = .13) were not significantly different between groups. However, the prevalence of LVH (defined as an increased LVMI) was higher in the test cohort (31.7% vs 19.2%, *P* < .05). LVH criteria as measured by ECG and echocardiography were stratified by gender as well (Table 2). There were no significant differences between male and female patients in both groups regarding abnormal ECG criteria. Also, although LVMI was increased in males as compared to females, prevalence of abnormal LVMI was comparable.

**Table 1:** Patient characteristics

	Test cohort (n = 167)	Validation cohort (n = 100)	*P*‐value
Age (years)	61 ± 13	48 ± 8	**<.001**
Female, n (%)	123 (74%)	70 (70%)	.49
Length (m)	1.67 ± 0.1	1.71 ± 0.1	.84
Weight (kg)	110 ± 15	127 ± 18	.27
BMI (kg/m^2^)	39 ± 4	43 ± 4	.20
BSA (m^2^)	2.3 ± 0.2	2.5 ± 0.2	**<.001**
Systolic BP (mmHg)	147 ± 25	142 ± 21	.09
Diastolic BP (mmHg)	76 ± 12	80 ± 12	.51
Heart rate (beats/min)	78 ± 16	71 ± 13	**.048**
Diabetes mellitus, n (%)	112 (67%)	21 (21%)	**<.001**
Hypertension, n (%)	80 (48%)	31 (31%)	**.008**
Left axis deviation, n (%)	16 (10%)	5 (5%)	.22
RaVL + SV3 (mm) (Cornell voltage)	11.8 ± 6	9.6 ± 5	**.002**
SV1 + RV5/RV6 (mm) (Sokolow‐Lyon index)	15.9 ± 6	15.7 ± 6	.81
SV4 + Sdeepest (mm) (Peguero‐Lo Presti criteria)	14.5 ± 5	12.1 ± 6	**.001**
LVM (g)	197 ± 67	196 ± 63	.83
LVM abnormal, n (%)	121 (72%)	69 (69%)	.63
LVMI (g/m^2^)	94 ± 81	79 ± 22	.13
LVMI abnormal, n (%)^a^	53 (32%)	19 (19%)	**.026**

*Note*: Values represent mean ± SD or n (%).

Abbreviations: BMI, body mass index; BP, blood pressure; BSA, body surface area; LVM, left ventricular mass; LVMI, left ventricular mass index.

a Used as the definition for LVH as defined by echocardiography.

**Table 2:** Criteria for LVH measured by ECG and echocardiography stratified by gender

	Test cohort (n = 167)			Validation cohort (n = 100)		
	Male (n = 44)	Female (n = 123)	*P* value	Male (n = 30)	Female (n = 70)	*P* value
ECG criteria						
Cornell voltage (mm)	14.8 ± 5.6	10.8 ± 5.6	**<.001**	11.1 ± 5.9	9.0 ± 0.4	.06
Cornell voltage abnormal, n (%)	3 (7%)	3 (2%)	.18	1 (3%)	1 (1%)	.37
Sokolow‐Lyon index (mm)	15.4 ± 5.5	1.73 ± 7.6	.09	15.5 ± 5.5	15.8 ± 6.1	.82
Solow‐Lyon abnormal, n (%)	1 (2%)	1 (1%)	.45	0	1 (1%)	.51
Peguero‐Lo Presti criteria (mm)	17.7 ± 5.8	13.4 ± 4.6	**<.001**	12.7 ± 6.4	11.8 ± 5.3	.45
Peguero‐Lo Presti abnormal n (%)	3 (7%)	4 (3%)	.31	2 (7%)	4 (6%)	.88
Echocardiography criteria						
LVM (g)	258 ± 80	174 ± 43	**<.001**	242 ± 56	176 ± 55	**<.001**
LVM abnormal, n (%)	36 (82%)	85 (69%)	.11	26 (87%)	43 (61%)	**.015**
LVMI (g/m^2^)	124 ± 130	84 ± 49	**.004**	92 ± 21	75 ± 26	**.002**
LVMI abnormal, n (%)	19 (43%)	34 (28%)	.06	5 (17%)	14 (20%)	.67

*Note*: Values represent mean ± SD or n (%).

Abbreviations: LVM, left ventricular mass; LVMI, left ventricular mass index.

### 3.2 Accuracy of established criteria for detection of LVH in obesity patients (test cohort)

The BMI adjusted Cornell voltage had the highest sensitivity (53% male, 32% female) followed by the Peguero‐Lo Presti criteria (16% male, 9% female). The Sokolow‐Lyon index had very poor sensitivity (0% male, 3% female) in this obese population (Table 3). On the other hand, the specificity of the criteria not multiplied by BMI was high (ranged from 96% to 100%), but relatively low for the BMI adjusted Cornell voltage (72% male, 85% female).

**Table 3:** Accuracy of the Cornell voltage, Sokolow‐Lyon index, Peguero‐Lo Presti criteria and the BMI adjusted Cornell voltage for detection of left ventricular hypertrophy in obesity patients using both the conventional cut‐off points and the adjusted criteria

Test Cohort	Gender	Sensitivity (95% CI)	Specificity (95% CI)	PPV (95% CI)	NPV (95% CI)
Conventional cut‐off points					
Cornell voltage	Male	16 (4‐40)	100 (83‐100)	100 (31‐100)	61 (45‐75)
	Female	3 (0‐17)	98 (91‐100)	33 (2‐87)	73 (63‐80)
Sokolow‐Lyon index	Male	0 (0‐21)	96 (78‐100)	0 (0‐95)	56 (40‐71)
	Female	3 (0‐17)	100 (95‐100)	100 (5‐100)	73 (64‐80)
Peguero‐Lo Presti criteria	Male	16 (4‐40)	100 (83‐100)	100 (31‐100)	61 (45‐75)
	Female	9 (2‐25)	99 (93‐100)	75 (22‐99)	74 (65‐81)
Cornell voltage * BMI^a^	Male	53 (29‐75)	72 (50‐87)	59 (33‐81)	67 (46‐83)
	Female	32 (2‐37)	85 (76‐92)	46 (26‐67)	80 (72‐87)
Adjusted Criteria					
Cornell voltage	Male	16 (4–40)	100 (83‐100)	100 (30‐100)	61 (45‐75)
	Female	6 (1‐21)	97 (90‐99)	40 (7‐83)	73 (64‐80)
Sokolow‐Lyon index	Male	21 (7‐46)	92 (72‐99)	67 (24‐94)	61 (43‐76)
	Female	18 (7‐35)	97 (90‐99)	67 (31‐910	75 (66‐82)
Peguero‐Lo Presti criteria	Male	32 (14‐57)	96 (78‐100)	86 (42‐99)	65 (47‐79)
	Female	24 (11‐42)	93 (85‐97)	57 (30‐81)	76 (67‐84)
Cornell voltage * BMI^a^	Male	47 (25‐70)	96 (78‐100)	90 (54‐99)	71 (52‐84)
	Female	9 (2‐25)	96 (88‐99)	43 (12‐80)	73 (64‐81)
Sokolow Lyon index * BMI^a^	Male	32 (14‐57)	96 (78‐100)	86 (42‐99)	65 (47‐79)
	Female	26 (14‐45)	93 (85‐97)	60 (33‐83)	77 (68‐84)
Peguero Lo Presti criteria * BMI^a^	Male	32 (14‐57)	96 (78‐100)	86 (42‐99)	65 (47‐79)
	Female	24 (11‐41)	96 (88‐99)	67 (35‐89)	77 (35‐89)
Validation Cohort					
Conventional cut‐off points					
Cornell voltage	Male	20 (1‐70)	100 (83‐100)	100 (5‐100)	86 (66‐95)
	Female	8 (0‐38)	100 (92‐100)	100 (5‐100)	82 (70‐90)
Sokolow‐Lyon index	Male	0 (0‐54)	100 (83‐100)	‐a	83 (64‐93)
	Female	0 (0‐28)	98 (89‐100)	0 (0‐95)	80 (68‐89)
Peguero‐Lo Presti criteria	Male	20 (1‐70)	96 (77‐100)	50 (3‐97)	85 (65‐95)
	Female	15 (3‐46)	96 (86‐100)	50 (9‐91)	82 (70‐90)
Cornell voltage * BMI^a^	Male	40 (7‐83)	88 (67‐98)	40 (7‐83)	88 (67‐97)
	Female	23 (6‐54)	87 (74‐94)	30 (8‐65)	82 (69‐91)
Adjusted criteria					
Cornell voltage	Male	20 (1–70)	100 (83‐100)	100 (5‐100)	86 (66‐95)
	Female	8 (4‐38)	98 (88‐100)	50 (3‐97)	81 (69‐90)
Sokolow‐Lyon index	Male	‐a	100 (83‐100)	‐a	83 (64‐93)
	Female	15 (2‐46)	90 (78‐96)	29 (5‐70)	81 (68‐90)
Peguero‐Lo Presti criteria	Male	20 (1–70)	96 (77‐100)	50 (3‐97)	85 (65‐95)
	Female	15 (3‐46)	94 (83‐99)	40 (7‐83)	82 (70‐90)
Cornell voltage * BMI^a^	Male	40 (7‐83)	92 (71‐99)	50 (9‐91)	88 (68‐97)
	Female	8 (0–38)	96 (86‐99)	33 (2‐87)	81 (69‐89)
Sokolow Lyon index * BMI^a^	Male	0 (0‐54)	92 (72‐99)	0 (0‐80)	81 (61‐93)
	Female	23(6–54)	83 (70‐91)	25 (7‐57)	81 (68‐90)
Peguero Lo Presti criteria * BMI^a^	Male	20 (1‐70)	96 (77–100)	50 (3‐97)	85 (65‐95)
	Female	23 (6–54)	92 (81‐98)	43 (12‐79)	83 (71‐91)

Abbreviations: BMI, body mass index; PPV, positive predictive value; NPV, negative predictive value.

a None of the male patients in the validation cohort had a positive Sokolow‐Lyon index.

### 3.3 Accuracy of adjusted criteria for detection of LVH in obesity patients (test cohort)

New cut‐off values for both males and females were defined for all criteria, with a fixed 95% specificity. The new cut‐off values for the Cornell voltage, Sokolow‐Lyon index, and Peguero‐Lo Presti criteria were, respectively, ≥20, 24, and 19 mm for females and ≥27, 27, and 23 mm for males. All criteria were multiplied by BMI. The optimal cut‐off values for the Cornell voltage*BMI, Sokolow‐Lyon index*BMI, and Peguero‐Lo Presti criteria*BMI were, respectively, ≥795, 885, and 780 mm*kg/m^2^ for females and ≥700, 984, and 900 mm*kg/m^2^ for males. Using these adjusted cut‐off values, the Cornell voltage*BMI, (RaVL+SV3)*BMI ≥700 mm*kg/m^2^, had the best sensitivity for males (47%, CI: 25%‐70%), specificity 96% (CI: 78%‐100%), ROC AUC 0.65, PPV 90% (CI: 54%‐99%), and NPV 71% (CI: 52%‐84%). The Sokolow‐Lyon index*BMI, (SV1 + RV5/RV6)*BMI≥885 mm*kg/m^2^, had the best sensitivity for females (26%, CI: 14%‐45%), specificity 93% (CI: 85%‐97%), ROC AUC 0.69, PPV 60% (CI: 33%‐83%), and NPV 77% (CI: 68%‐84%).

### 3.4 Prospective validation of the adjusted criteria for detection of LVH in obesity patients (validation cohort)

When the new criteria were tested in the validation cohort, again the adjusted Cornell voltage*BMI had the best sensitivity for males (40%, CI: 7%‐83%), specificity 92% (CI: 71%‐99%), ROC AUC 0.78, PPV 50% (CI: 9%‐91%), and NPV 88% (CI: 68%‐97%). The Sokolow‐Lyon index*BMI again had the best sensitivity for females (23%, CI: 6%‐54%), specificity 83% (CI: 70%‐91%), ROC AUC 0.57, PPV 25% (CI: 7‐57%), and NPV 81% (CI: 68%‐90%). None of the male patients in the validation cohort had a positive Sokolow‐Lyon index at a cut‐off value of 27 mm. There were no substantial differences between the sensitivity and specificity in the test cohort and validation cohort.

## 4 DISCUSSION

In the current study, we demonstrated that in obesity patients, established ECG criteria for the detection of LVH lack sufficient sensitivity for application in daily clinical practice. We propose new criteria, (RaVL+SV3)*BMI≥700 mm*kg/m^2^ for males and (SV1 + RV5/RV6)*BMI≥885 mm*kg/m^2^ for females, for the detection of LVH in obesity patients with improved sensitivity, without losing specificity.

The explanation of the poor sensitivity of the established ECG criteria for detection of LVH (3%‐9% in females and 0%‐16% in males) may be that obesity patients commonly have reduced voltages in the precordial ECG leads, probably because the ECG voltages at the skin level are attenuated by the subcutaneous adipose tissue.^14^ The sensitivity of these criteria may be improved by adjustment of the cut‐off values and correction for BMI. Applying this, the Cornell voltage*BMI for males and Sokolow‐Lyon index*BMI for females, showed an important improvement of the sensitivity of an ECG for the detection of LVH in obesity patients to 47% and 26%, respectively, using the optimal cut‐off values (both identified in analysis using a fixed specificity of 95%).

Because in obesity patients with cardiac disease there may often already be a clinical indication for an echocardiogram, an ECG as a screening tool for detection of LVH may have the most value in obesity patients without known cardiac disease. In the current study for the first time, adjusted ECG criteria for the detection of LVH were tested in such a relatively low‐risk obese population. Even in these subjects, the proposed new criteria performed fairly very well (sensitivity of 40% for the Cornell voltage*BMI in males and 23% for the Sokolow‐Lyon index*BMI in females, using the optimal cut‐off values). Although these sensitivity values appear to be rather poor, also in lean subjects the sensitivity of ECG criteria for LVH is known to be limited. A review of multiple studies in different healthcare settings found that the sensitivity of the Cornell voltage and Sokolow‐Lyon index ranged from 2% to 52% with a specificity ranging from 71% to 100%.^24^ Therefore, in our study, it was shown that the sensitivity of an ECG to detect LVH in obesity patients without known cardiac disease may be comparable to known sensitivity in lean subjects when using the proposed new criteria.

When adjusting the Cornell voltage by BMI as designed by Angeli et al.,^17^ the sensitivity of an ECG to detect LVH increased even to 53%; however, the specificity decreased to 72%. In previous studies,^10^, ^14^, ^22^, ^23^ a fixed specificity level of 95% was chosen because this is supposed to be sufficient to render an ECG a cost‐effective alternative to echocardiography in screening populations for the presence of LVH. Therefore, we also used this 95% fixed specificity and identified optimal sensitivity values by adjusting the cut‐off values of the criteria. Moreover, in the study by Angeli et al., this criterion was not specifically tested in a group of obese patients. The mean BMI in their cohort was 26.7 kg/m^2^, which is much lower than the mean BMI of our test cohort and validation cohort (39 and 43 kg/m^2^, respectively).

Some other studies regarding the optimization of ECG criteria for the detection of LVH in obesity patients have been performed before. Rider et al. made an adjustment to the cut‐off value of the Sokolow‐Lyon index (+8 mm). This improved the sensitivity to 27% (specificity 93%) in their test cohort and 25% in their validation cohort.^25^ Also, Robinson et al. designed a new criterion [RaVL + (BMI − 29) × 0.017], which improved the sensitivity to 42%, however, with a relatively decreased specificity of 83%.^12^ Finally, Rodrigues et al. made an adjustment to the Cornell voltage (cut‐off value ≥27 mm) which improved the sensitivity to 21% with a specificity of 95%.^23^ Nevertheless, in none of these studies, the sensitivity of the established ECG criteria for the detection of LVH in lean subjects was approached.

All adjusted criteria in our study had better sensitivities in males than in females. This difference is possibly because of the abundant breast tissue in obese women,^26^ which may also explain why the Cornell voltage performed relatively poor in women (Table 3). The positioning of lead V3, an important lead for the Cornell voltage, is usually on a location of relatively plentiful breast tissue as compared to the position of V1 and V5 used for the Sokolow‐Lyon index, which appeared to be the best performing criterion in female obesity patients. An explanation could also be that in general women have a smaller LVM (in our obese population 175 ± 48 g in females vs 252 ± 72 g in males, *P* < .001) leading to smaller S wave amplitude in V3, which measures posteriorly directed myocardial electrical activity.^9^, ^27^

Finally, it may seem difficult to implement our proposed criteria into daily practice since, apart from the necessity to assess BMI, it would require the use of different criteria in males and females. However, nowadays ECG devices already use programmed algorithms for standard interpretation. It will be a relatively minor issue to add our proposed new criteria to these modern devices, allowing easy clinical use without extra effort.

### 4.1 Limitations

LVM was estimated by 2D echocardiography, despite reports demonstrating superior accuracy of cardiac magnetic resonance imaging, especially in obesity patients.^28^, ^29^ However, echocardiography is known to have good reproducibility for the diagnosis of LVH and remains the most frequently used method in clinical practice.^30^ Also, LVH diagnosed by ECG is known to be a marker of adverse electric remodeling even without LVH diagnosed by echocardiography. Thus, also without association with echocardiographic LVH, some ECG criteria may still be associated with prognosis.^31^, ^32^ Although obesity is usually defined as BMI ≥30 kg/m^2^, all patients included in our study had a BMI ≥35 kg/m^2^ because this was an inclusion criterion for the CARDIOBESE study. Therefore, the conclusions may only be applied to morbidly obese patients and not to obesity patients in general. The sample size was relatively small. The validation cohort had a relatively low prevalence of LVH; therefore, the PPV values even for the new criteria were low. However, as mentioned before, in the current study for the first time, adjusted ECG criteria for the detection of LVH were validated in a relatively low‐risk obese population without known or suspicion of cardiovascular disease. Racial differences in the diagnosis of LVH were not addressed in this study. Also, the abilities of the proposed criteria to predict outcomes (eg, incident cardiovascular morbidity) are not known. Finally, we included only BMI as an obesity index and could not assess whether, for example, waist circumference or epicardial fat thickness is superior to BMI to adjust the voltage ECG criteria.

## 5 CONCLUSIONS

Established ECG criteria for the detection of LVH lack sufficient sensitivity in obesity patients. We propose new criteria, (RaVL+SV3)*BMI ≥700 mm*kg/m^2^ for males and (SV1 + RV5/RV6)*BMI ≥885 mm*kg/m^2^ for females, for the detection of LVH in obesity patients with improved sensitivity (47% in males and 26% in females), approaching known sensitivity of the most commonly used ECG criteria in lean subjects.

## CONFLICT OF INTEREST

None declared.
